# Characteristics and influencing factors of neuroendocrine dysfunction in patients with adult-onset craniopharyngioma

**DOI:** 10.3389/fendo.2025.1615720

**Published:** 2025-09-15

**Authors:** Ying Guo, Songbai Gui, Pinan Liu, Yazhuo Zhang, Liyong Zhong, Jian Xu

**Affiliations:** ^1^ Department of Endocrinology, Beijing Tiantan Hospital, Capital Medical University, Beijing, China; ^2^ Department of Neurosurgery, Beijing Tiantan Hospital, Capital Medical University, Beijing, China; ^3^ Beijing Neurosurgical Institute, Capital Medical University, Beijing, China

**Keywords:** adamantinomatous craniopharyngioma, papillary craniopharyngioma, adenohypophyseal dysfunction, diabetes insipidus, clinical prognosis

## Abstract

**Objective:**

The aim of the study was to compare neuroendocrine dysfunction patterns in adult-onset adamantinomatous craniopharyngiomas (ACPs) and papillary craniopharyngiomas (PCPs) before and after surgery, and identify factors that influence neuroendocrine outcomes in these two histopathological types.

**Methods:**

A retrospective analysis was conducted on 390 patients with adult-onset craniopharyngioma (CP) patients, including 272 patients with ACP and 118 patients with PCP. The pre- and post-operative neuroendocrine parameters were compared, and the factors that contributed to poor endocrine outcomes were identified.

**Results:**

Suprasellar tumor extension (83.1% *vs.* 70.6%, *p* = 0.01), mass effects (81.4% *vs.* 68.4%, *p*<0.01), and pre-operative central diabetes insipidus (CDI; 35.6% *vs.* 21.0%, *p* = 0.02) rates were higher in the PCP group, when compared to the ACP group. However, both PCP and ACP patients presented with a post-operative increase in growth hormone deficiency (GHD), CDI, and hypothalamic-pituitary-target dysfunction (*p*<0.05 *vs.* pre-operative baselines). Furthermore, the hypothalamic-pituitary-adrenal (HPA) axis dysfunction (75.4% *vs.* 65.3%, *p* = 0.04) and GHD (50.0% *vs.* 34.7%, *p*<0.01) rates were higher in the ACP group, when compared to the PCP group. Surgical intervention had a greater detrimental effect on overall pituitary function in ACP patients, when compared to PCP patients. The ACP pathological type, larger tumors, and milder pre-operative endocrine dysfunction were associated with a significantly higher risk of postoperative pituitary hormone deficiencies (*p*<0.05).

**Conclusions:**

Surgical intervention may exacerbate pituitary dysfunction in adult patients with ACP and PCP, although different factors influence the adverse endocrine outcomes for these two pathological types.

## 1 Introduction

Craniopharyngioma (CP) is a benign neoplasm of the central nervous system that is located in the sellar or parasellar regions. Adamantinomatous craniopharyngioma (ACP) originates from the epithelial remnants of Rathke’s pouch or embryonic craniopharyngeal duct, while papillary craniopharyngioma (PCP) is derived from the metaplastic squamous epithelium of the primitive stomodeum (1). These two histopathological types have distinct tumorigenic, radiological, histopathological, genetic, and methylation features (2). Patients with CPs typically present mass effect symptoms, such as headaches and visual disturbances, along with concomitant anterior pituitary dysfunction and hypothalamic syndrome (3, 4). Neuroendocrine dysfunction in these patients may be exacerbated by the destructive effects of tumor growth in the hypothalamic-pituitary region, and the iatrogenic damage from surgical intervention or radiotherapy, resulting in a significant reduction in long-term quality of life (QOL) (3, 5).

Previous studies have revealed significant heterogeneity in the functional status of the hypothalamic-pituitary axes in CP patients, which can be attributed to the age at onset, pathological type, tumor size/location, therapeutic approach, and recurrence status (6–8). The aim of the present study was to compare the pre-operative and post-operative patterns of neuroendocrine dysfunction in patients with adult-onset ACP and PCP, and identify the potential factors that influence the neuroendocrine outcomes of these two histopathological variants.

## 2 Subjects and methods

### 2.1 Study population

A total of 390 adult patients with CP, who underwent initial tumor resection surgery at Beijing Tiantan Hospital, Capital Medical University from 2015 to 2022, were enrolled. Based on the histopathological findings, these patients were stratified into the ACP group (*n* = 272) and PCP group (*n* = 118). During screening, patients who (1) received prior surgical treatment for CP (at our institution or elsewhere), (2) lacked pre- or post-operative endocrine evaluations, (3) had inconclusive pathological diagnoses, or (4) exhibited concurrent primary hypothyroidism, adrenal insufficiency, hypogonadism, or other intracranial neoplasms were excluded.

### 2.2 Data collection

The present study is a single-center, retrospective cohort study. The baseline data of the patients and surgery-related indicators were collected, which included age at onset, gender, tumor size and location, pathological type, surgical approach, extent of resection (gross total resection or not), and pre-operative endocrine function status. Pre- and post-surgery pituitary dysfunction was evaluated for the ACP and PCP groups according to the deficiencies in four adenohypophyseal hormones, antidiuretic hormone deficiency, and hyperprolactinemia (**Table 1**). The risk factors influencing the post-operative pituitary function in ACP and PCP patients were analyzed.

**Table 1:** Criteria for evaluating CDI and pituitary deficiencies in patients with CP (4, 9–11).

Indices	Diagnostic criteria	Score
CDI	Urine volume >4,000 mL/24 hours, urine specific gravity <1.005. The results of the water deprivation test with vasopressin challenge confirmed the diagnosis of CDI.	1
HPA	Based on the absence of glucocorticoid drug usage and the presence of clinical symptoms, such as fatigue, anorexia, hyponatremia, etc., the serum concentrations of COR and ACTH should meet one of the following two criteria: (1) COR <5 μg/dL and ACTH <25.0 pmol/L at 8 am on two separate occasions, (2) COR <5 μg/dL and ACTH <25.0 pmol/L at 8 am once, with disappearance of COR/ACTH circadian rhythm^a^.	1
HPT	FT4 is lower than the reference range, accompanied by normal, decreased, or slightly increased TSH levels (<10 mU/L) twice, or once combined with symptoms and signs of hypothyroidism.	1
HPG	Men: decreased T levels; decreased FSH and LH levels or levels at the lower limit of normal values. Premenopausal women: decreased E2 levels; decreased FSH and LH levels or levels at the lower limit of normal values. Postmenopausal women: decreased E2 levels; no increase in FSH and LH levels.	1
GHD	IGF-1 and IGFBP-3 are below the normal range for age and gender matching controls, accompanied by at least one other adenohypophysis hypofunction, or at least one of the GH stimulation tests with the peak value of GH <5 μg/L.	1
Hyperprolactinemia	Serum prolactin levels above the normal range (>20 ng/mL in men and postmenopausal women, and >30 ng/mL in premenopausal women)	0

a Disappearance of COR/ACTH circadian rhythm: Serum concentration of COR <5.00 μg/dL and ACTH <25.00 pmol/L at 8 am, COR <2.50 μg/dL and ACTH <15.00 pmol/L at 4 pm, and COR <2.50 μg/dL and ACTH <15.00 pmol/L at 12 am. CDI, central diabetes insipidus; CP, craniopharyngioma; HPA, hypothalamus-pituitary-adrenal axis; COR, cortisol; ACTH, adrenocorticotrophic hormone; HPT, hypothalamus-pituitary-thyroid axis; FT4, free thyroxine; TSH, thyroid stimulating hormone; HPG, hypothalamus-pituitary-gonad axis; T, testosterone; FSH, follicle-stimulating hormone; LH, luteinizing hormone; E2, estradiol; GHD, growth hormone deficiency; IGF-1, insulin-like growth factor-1; IGFBP-3, insulin-like growth factor-binding protein 3; GH, growth hormone.

### 2.3 Data analysis

Measurement data that conformed to normal distribution have been presented as mean ± standard deviation (SD), and were compared using Student’s *t*-test. Non-normally distributed data have been presented as median (interquartile range), and were compared using Mann-Whitney *U*-test. Categorical data have been presented as frequencies (*n*) and percentages (%), and were compared by the Chi-squared test. Univariate and multivariate logistic regression analyses were performed to identify factors associated with aggravated endocrine dysfunction. A *p*-value of < 0.05 indicated statistical significance, unless stated otherwise. The data were analyzed using the SPSS Statistics 25 software (SPSS Inc.).

## 3 Results

### 3.1 Baseline characteristics of patients

**Figure 1:**
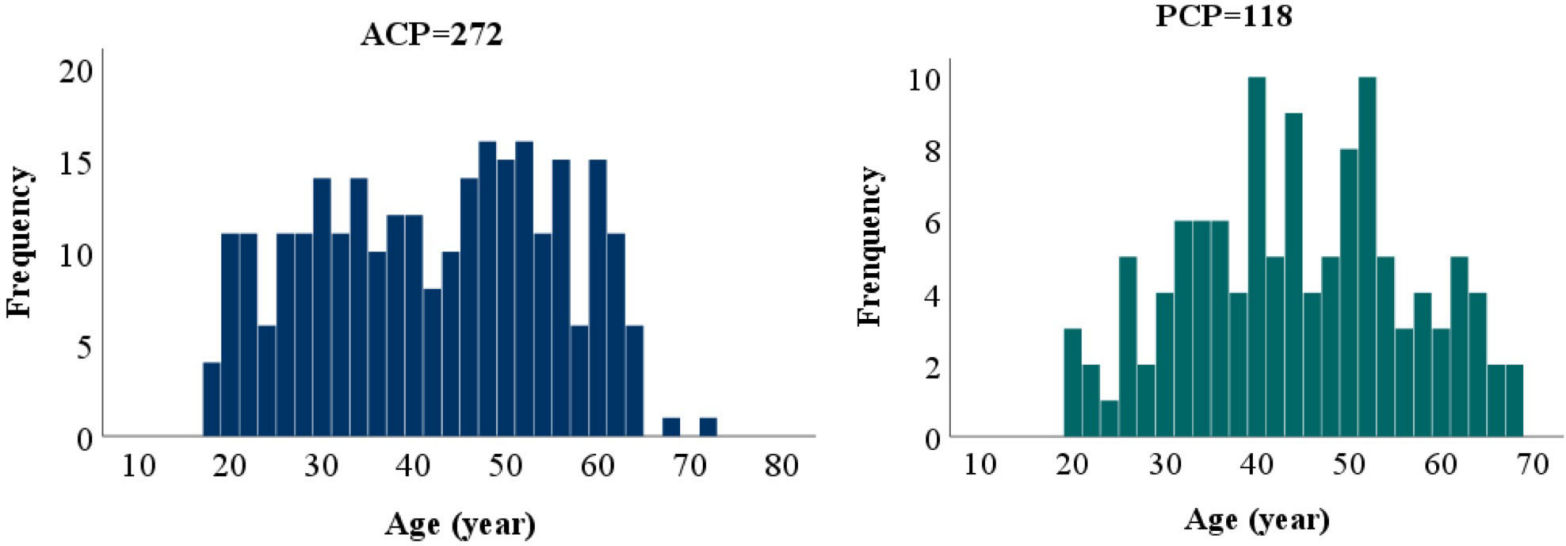
Age distribution of the ACP and PCP groups.

Adult patients (≥18 years old) with CP, who underwent first-time tumor resection at the Department of Neurosurgery, Beijing Tiantan Hospital between 2015 and 2022, were enrolled for the present study. There were 390 patients in the final cohort, including 272 patients with ACP and 118 patients with PCP. The male-to-female (M/F) ratio was 1.2:1.0 for the entire cohort, 1.0:1.0 for the ACP group, and 1.9:1.0 for the PCP group, with a significantly higher proportion of males in the latter (*p*<0.01). Age was distributed non-normally in the ACP group, and normally in the PCP group. No significant difference in age distribution was observed between the two groups (**Figure 1**).

The proportion of patients who presented with typical tumor mass effect symptoms, such as headaches, visual/field deficits and vomiting, before surgery was significantly higher in the PCP group (81.4%), when compared to the ACP group (68.4%) (*p*<0.01). In addition, the prevalence of suprasellar tumor localization was higher in the PCP group, when compared to the ACP group (83.1% *vs.* 70.6%, *p* = 0.01). However, no significant differences were observed between the two groups in terms of the extent of resection (GTR/STR), surgical method (transsphenoidal/transcranial), tumor volume, or duration of post-operative follow-up.

The median follow-up duration for the CP patients was 12 months (3-24 months). Tumor recurrence occurred in 80 patients (20.5%), the median time to recurrence was 24 months (14-36 months), and the mean recurrent tumor diameter was 2.85 cm (± 1.22). Among the recurrent cases, 52.5% (42/80) underwent secondary surgical resection, while 26.3% (21/80) received radiotherapy. The recurrence rate, time to recurrence, tumor size, and treatment modality were similar in both groups (**Table 2**).

**Table 2:** Demographic and clinical characteristics at baseline.

Variables	CP total (*n* = 390)	ACP group (*n* = 272)	PCP group (*n* = 118)	*P*-value (ACP *vs.* PCP)
Age (years)	43.00 (32.00, 52.00)	43.00 (30.25, 52.00)	43.86 (12.02)	0.17
Gender (Male/Female)	215/175	138/134	77/41	<0.01
Tumor mass effects^a^ (Yes/No, %)	282/108 (72.3%)	186/86 (68.4%)	96/22 (81.4%)	<0.01
Resection (GTR^b^/STR^c^)	318/72	220/52	98/20	0.61
Surgical method (Transsphenoidal/Transcranial)	101/289	75/197	26/92	0.25
Tumor position (suprasellar/sellar region, %)	290/100 (74.4%)	192/80 (70.6%)	98/20 (83.1%)	0.01
Tumor volume (cm^3^)^d^	7.95 (4.00, 16.93)	8.00 (4.10, 17.88)	7.80 (3.60, 15.80)	0.49
Mean diameter of tumor (cm)	2.6 (2.0, 3.3)	2.6 (2.1, 3.3)	2.5 (2.0, 3.2)	0.46
Follow-up (months)	12 (3, 24)	12 (3, 24)	12 (3, 24)	0.58
Recurrence^e^ or Progression^f^ (*n*, %)	80, 20.51%	58, 21.32%	22, 18.64%	0.55
Time of tumor recurrence/progression (months)	24 (14, 36)	24 (16, 36)	19 (12, 36)	0.40
Average diameter of recurrent/progressive tumor (cm)	2.85 (1.22)	2.95 (1.30)	2.58 (0.99)	0.38
Re-operation (*n*, %)	42, 52.50% (42/80)	32, 55.17% (32/58)	10, 45.45% (10/22)	0.44
Radiotherapy (*n*, %)	21, 26.25% (21/80)	12, 20.69% (12/58)	9, 40.91% (9/22)	0.07

CP, craniopharyngioma; ACP, adamantinomatous craniopharyngioma; PCP, papillary craniopharyngiomas; ^a^Tumor mass effects: patient presenting at least one of the following three symptoms: intracranial hypertension (headache or nausea), vision loss, and visual-field defect; ^b^GTR, gross-total resection; ^c^STR, sub-total resection; ^d^Tumor volume = Length × Width × Height × 0.50; ^e^Recurrence: resurgence of tumor markers on the postoperative MRI of patients who underwent GTR; ^f^Progression: an increase in residual tumor volume indicated by MRI during follow-up in patients who underwent STR.

### 3.2 Pituitary-target axes dysfunction

**Figure 2:**
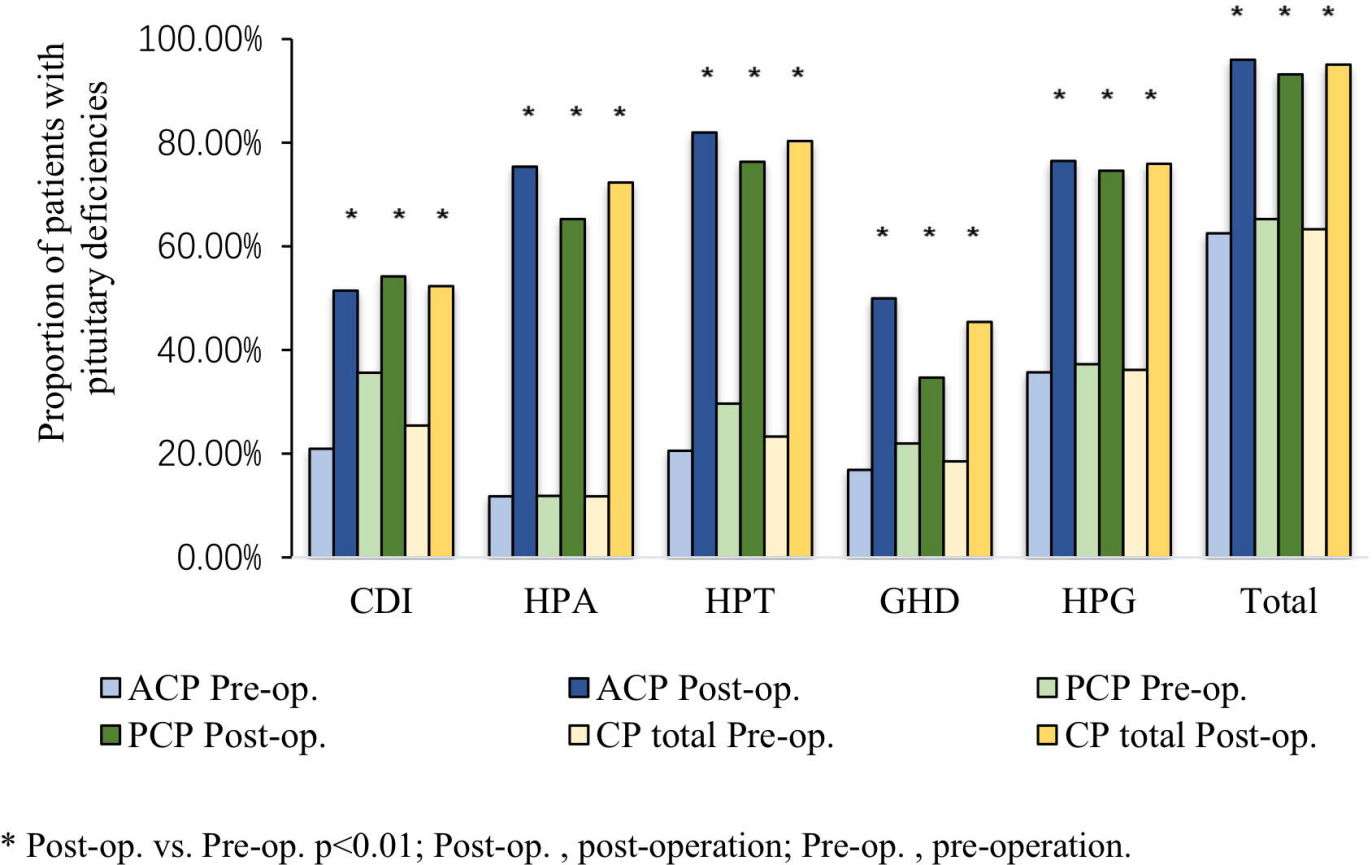
Comparison of pre- and post-operative hypothalamic-pituitary hormone deficiencies in adult CP patients.

Pituitary function was assessed for all CP patients before surgery and at the last follow-up using the evaluation criteria outlined in **Table 1**. Pre-operative findings: The proportion of patients with central diabetes insipidus (CDI) was significantly higher in the PCP group, when compared to the ACP group (35.6% *vs.* 21.0%, *p* = 0.02). However, no significant differences were observed in the proportion of patients with dysfunctional hypothalamic-pituitary-adrenal (HPA), hypothalamic-pituitary-thyroid (HPT), or hypothalamic-pituitary-gonadal (HPG) axis (11.8%, 23.3% and 36.2% respectively), growth hormone deficiency (GHD, 18.5%), or hyperprolactinemia (45.1%). Post-operative outcomes: The incidence of HPA, HPT and HPG axes dysfunction, GHD, and CDI significantly increased in the entire cohort, and in the ACP and PCP subgroups, after a median follow-up of 12 months, when compared to pre-operative baselines (**Figure 2**). However, the prevalence of hyperprolactinemia did not present with any significant post-operative changes in any group. Notably, the post-operative rates of HPA axis impairment (75.4% *vs.* 65.3%, *p* = 0.04) and GHD (50.0% *vs.* 34.7%, *p*<0.01) were higher in the ACP group, when compared to the PCP group (**Table 3**).

**Table 3:** Characteristics of hypothalamic-pituitary dysfunction in patients with CP before and after surgery.

Variables	CP total (*n* = 390)			ACP group (*n* = 272)			PCP group (*n* = 118)			Pre-operation *p*-value (ACP-PCP)	Post-operation *p*-value (ACP-PCP)
	Pre-operation	Post-operation	*P*-value	Pre-operation	Post-operation	*P*-value	Pre-operation	Post-operation	*P*-value		
CDI (*n*, %)	99, 25.4%	204, 52.3%	<0.01	57, 21.0%	140, 51.5%	<0.01	42, 35.6%	64, 54.2%	<0.01	0.02	0.62
HPA (*n*, %)	46, 11.8%	282, 72.3%	<0.01	32, 11.8%	205, 75.4%	<0.01	14, 11.9%	77, 65.3%	<0.01	0.98	0.04
HPT (*n*, %)	91, 23.3%	313, 80.3%	<0.01	56, 20.6%	223, 82.0%	<0.01	35, 29.7%	90, 76.3%	<0.01	0.05	0.19
GHD (*n*, %)	72, 18.5%	177, 45.4%	<0.01	46, 16.9%	136, 50.0%	<0.01	26, 22.0%	41, 34.7%	0.03	0.23	<0.01
HPG (*n*, %)	141, 36.2%	296, 75.9%	<0.01	97, 35.7%	208, 76.5%	<0.01	44, 37.3%	88, 74.6%	<0.01	0.76	0.69
Hyperprolactinemia (*n*, %)	176, 45.1%	190, 48.7%	0.32	121, 44.5%	133, 48.9%	0.30	55, 46.6%	57, 48.3%	0.79	0.70	0.91
Pituitary Deficiencies total (n,%)	247, 63.3%	371, 95.1%	<0.01	170, 62.5%	261, 96.0%	<0.01	77, 65.3%	110, 93.2%	<0.01	0.60	0.25

CP, craniopharyngioma; ACP, adamantinomatous craniopharyngioma; PCP, papillary craniopharyngiomas; CDI, central diabetes insipidus; HPA, hypothalamic-pituitary-adrenal; HPT, hypothalamic-pituitary-thyroid; GHD, growth hormone deficiency; HPG, hypothalamic-pituitary-gonadal.

### 3.3 Degree of pituitary dysfunction

To evaluate the severity of pituitary dysfunction in CP patients, we quantified the cumulative number of deficiencies in anterior and posterior pituitary hormones, including the adrenocorticotropic hormone (ACTH), thyroid-stimulating hormone (TSH), growth hormone (GH), gonadotropins (LH/FSH), and antidiuretic hormone (ADH). The parameters for scoring the pituitary function are listed in **Table 1**. Each indicator of hormonal dysfunction (*e.g.* ACTH deficiency) was assigned 1 point, while normal function was assigned 0 point. A higher total score reflected more severe pituitary impairment due to deficiencies in multiple hormones.

**Figure 3:**
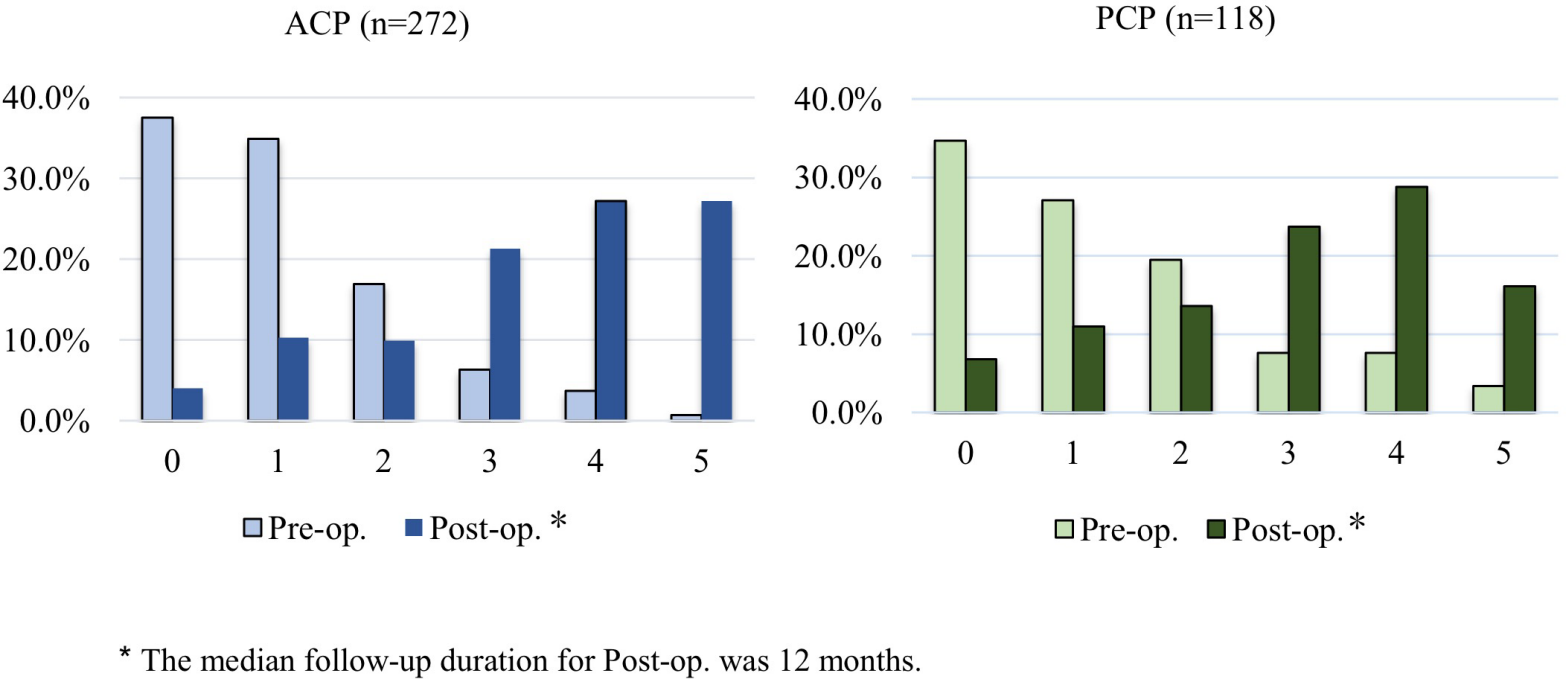
Comparison of pre- and post-operative hormone deficiencies between theACP and PCP groups.

The pre-operative and post-operative status of ACTH, TSH, GH, LH/FSH, and ADH deficiencies in the entire cohort and the subgroups are summarized in **Table 4**. The severity of pituitary hormone deficiencies significantly increased after surgery in the ACP and PCP groups, when compared to the respective pre-operative baselines (*p*<0.01). Notably, there was a more pronounced deterioration in the ACP group, when compared to the PCP group (*p*<0.01). This indicates that the surgical intervention significantly exacerbated the neuroendocrine dysfunction associated with CP, with disproportionately severe pituitary impairment in patients with ACP (**Figure 3**). Furthermore, we also investigated the impact of tumor location on the severity of preoperative endocrine hormone deficiencies in the ACP and PCP patients. In the ACP cohort, patients with sellar tumors exhibited significantly higher levels of hormone deficiency compared to those with suprasellar tumors (*p* = 0.02). Due to the limited sample size of sellar PCP cases (*n* = 20), no statistically significant difference in endocrine dysfunction severity was observed between the subgroups demarcated based on tumor location. However, the proportion of PCP patients with deficiencies in ≥ 4 hormone axes was substantially higher in the sellar group than in the suprasellar group (15.0% *vs.* 10.2%; **Table 4**).

**Table 4:** Comparison of severity in endocrine hormone deficiencies between ACP and PCP.

Number of hormone deficits	CP total (*n* = 390)				ACP group (*n* = 272)				PCP group (*n* = 118)				Pre-operation							
	Pre-operation		Post-operation		Pre-operation		Post-operation		Pre-operation		Post-operation		ACP group (*n* = 272)				PCP group (*n* = 118)			
													Suprasellar (*n* = 192)		Sellar (*n* = 80)		Suprasellar (*n* = 98)		Sellar (*n* = 20)	
0	143	36.7%	19	4.9%	102	37.5%	11	4.0%	41	34.7%	8	6.8%	79	41.1%	23	28.8%	33	33.7%	8	40%
1	127	32.6%	41	10.5%	95	34.9%	28	10.3%	32	27.1%	13	11.0%	68	35.4%	27	33.8%	27	27.6%	5	24%
2	69	17.7%	43	11.0%	46	16.9%	27	9.9%	23	19.5%	16	13.6%	26	13.5%	20	25%	20	20.4%	3	15%
3	26	6.7%	86	22.1%	17	6.3%	58	21.3%	9	7.6%	28	23.7%	13	6.8%	4	5.0%	8	8.2%	1	5%
4	19	4.9%	118	30.3%	10	3.7%	74	27.2%	9	7.6%	34	28.8%	5	2.6%	5	6.3%	8	8.2%	1	5%
5	6	1.5%	83	21.3%	2	0.7%	74	27.2%	4	3.4%	19	16.1%	1	0.5%	1	1.3%	2	2.0%	2	10%
	*p*<0.01				*p*<0.01				*p*<0.01				*p* = 0.02				*p* = 0.75			

CP, craniopharyngioma; ACP, adamantinomatous craniopharyngioma; PCP, papillary craniopharyngiomas; The Wilcoxon signed-rank test was used for the intra-group comparisons of the number of hormone deficits before and after surgery in each group; The Mann-Whitney U-test was used to compare the degree of postoperative impairment of neuroendocrine function between the ACP and PCP groups, *p*<0.01.

To evaluate the post-operative changes in the severity of pituitary hormone deficiency for each patient group, the pre-operative cumulative number of pituitary hormone deficiencies was subtracted from the corresponding post-operative counts. The outcomes were categorized, as follows: improvement, no change, or deterioration. The improvement or deterioration in pituitary hormone deficiencies was further stratified according to the magnitude of decrease or increase (**Table 5**). Negative scores indicated reduced post-operative pituitary hormone deficiencies, when compared to the pre-operative status. Furthermore, a zero-score indicated no change, while positive scores indicated more severe post-operative deficiencies.

**Table 5:** Changes in frequency of pituitary deficiencies at post-operation, when compared to pre-operative baseline.

Changes in number of pituitary deficiencies	CP total		ACP group		PCP group		*P*-value (ACP *vs.* PCP)
	*n*	*n*/390 (%)	*n*	*n*/272 (%)	*n*	*n*/118 (%)	
Alleviation	19	4.9%	10	3.7%	9	7.6%	0.10
-2	3	0.8%	1	0.4%	2	1.7%	–
-1	16	4.1%	9	3.3%	7	5.9%	–
Unchanged, 0	44	11.3%	26	9.6%	18	15.3%	0.10
Aggravation	327	83.8%	236	86.7%	91	77.1%	0.02
1	80	20.5%	52	19.1%	28	23.7%	–
2	77	19.7%	50	18.4%	27	22.9%	–
3	87	22.3%	67	24.6%	20	16.9%	–
4	65	16.7%	53	19.5%	12	10.2%	–
5	18	4.6%	14	5.1%	4	3.4%	–

CP, craniopharyngioma; ACP, adamantinomatous craniopharyngioma; PCP, papillary craniopharyngiomas; Changes in the number of pituitary deficiencies = The number of pituitary hormone deficiencies after surgery - The number of pituitary hormone deficiencies before surgery. -2 to -1: the number of disorders decreased after surgery; 0: no change; 1 to 5: the number of disorders increased after surgery.

Pituitary function improvement was observed in 3.7% of the ACP patients, when compared to 7.6% of PCP patients (*p* = 0.10). In addition, 9.6% of ACP patients maintained stable hormone function, as opposed to 15.3% of PCP patients (*p* = 0.10). Finally, 86.7% of ACP patients experienced functional deterioration, when compared to 77.1% of PCP patients (*p* = 0.02). These findings suggest that surgical intervention exacerbates pituitary dysfunction in ACP patients relative to PCP patients.

### 3.4 Risk factors of post-surgery neuroendocrine dysfunction

The present findings suggest that surgical treatment can significantly exacerbate hypothalamic-pituitary dysfunction in adult patients with CP. However, since surgery remains the primary therapeutic option for CP, it is imperative to identify risk factors that influence post-operative neuroendocrine function in adult patients with ACP and PCP. To this end, factors (apart from surgery) that may contribute to poor neuroendocrine outcomes in these patients were screened using the post-operative neuroendocrine function score as the dependent variable (worsened = 1, unchanged or improved = 0). Univariate logistic regression was initially performed to assess the correlation between the outcome and 10 variables, including age at onset, gender, tumor volume, tumor location, extent of resection (GTR *vs.* STR), surgical method (transsphenoidal *vs.* transcranial), presence of mass effect, histopathological type (ACP *vs.* PCP), tumor recurrence/progression, and pre-operative neuroendocrine function score. As shown in **Table 6**, the ACP pathological type, larger tumor volume, and lower pre-operative pituitary function score were associated with increased risk of worsened post-operative neuroendocrine function (*p*<0.20). Furthermore, pathological classification, tumor volume, and pre-operative neuroendocrine dysfunction severity were identified as independent risk factors for aggravated post-operative endocrine dysfunction in adult CP patients (*p*<0.05) after adjusting for confounders in the multivariate logistic regression analysis. Specifically, the ACP pathological type was associated with a 1.51-fold higher risk of worse post-operative pituitary hormone deficiencies, when compared to PCP (*p* = 0.04). In addition, every 1 cm^3^ increase in tumor volume increased the risk of post-operative pituitary hormone deficiencies by 5% (*p*<0.01), while every 1-point decrease in pre-operative neuroendocrine function score increased the risk by 48% (*p*<0.01). No significant associations were observed between post-operative endocrine deterioration, and gender, age, tumor mass effect, surgical approach, extent of resection, tumor location, or recurrence/progression status (**Table 6**).

**Table 6:** Risk factors for aggravated hormonal deficiency in CP patients after surgery.

Parameters	Univariate		Multivariate	
	OR (95% CI)	*p*-value	OR (95% CI)	*p*-value
Gender (Male/Female)	0.84 (0.49, 1.45)	0.53	0.96 (0.52, 1.78)	0.90
Age of onset (Years)	1.00 (0.98, 1.02)	0.92	1.01 (0.99, 1.04)	0.23
Tumor mass effects (Yes/No)	0.71 (0.37, 1.34)	0.29	0.78 (0.39, 1.57)	0.48
Surgical method (Transcranial/Transsphenoidal)	1.07 (0.58, 1.97)	0.83	0.87 (0.43, 1.75)	0.69
Resection (GTR/partial)	0.92 (0.46, 1.87)	0.82	0.77(0.33, 1.77)	0.53
Classification (ACP/PCP)	1.95 (1.12, 3.39)	0.02	1.51 (1.05, 2.83)	0.04
Recurrence (Yes/No)	1.44 (0.70, 2.98)	0.32	1.65 (0.72, 3.78)	0.23
Tumor volume (cm^3^)	1.03 (1.00, 1.06)	0.07	1.05 (1.01, 1.08)	<0.01
Tumor location (Suprasellar/Sellar region)	1.09 (0.59, 2.00)	0.79	0.95 (0.46, 1.97)	0.89
Number of hormone deficits at pre-operation	0.52 (0.42, 0.65)	<0.01	0.48 (0.38, 0.60)	<0.01

CP, craniopharyngioma; OR, odds ratio; 95% CI, 95% confidence interval; GTR, gross-total resection; ACP, adamantinomatous craniopharyngioma; PCP, papillary craniopharyngioma.

## 4 Discussion

CPs frequently compress or infiltrate the hypothalamic-pituitary axes, resulting in complex endocrine disorders that significantly impair a patient’s QOL (12, 13). Despite increased post-operative endocrine surveillance in adult CP patients in recent years (14, 15), little is known regarding the differences in endocrine dysfunction between these two pathological types. In the present cohort, the prevalence of ACP was significantly higher than that of PCP, which is consistent with previous reports (4, 16). Furthermore, there was no significant predilection towards either gender in the ACP group, whereas the PCP group had a higher proportion of male patients. These findings align with the gender distribution patterns reported by Feng et al. (17), but contradict historical data that suggest equal gender distribution in CP cases (16).

Adult-onset CP primarily manifests as intracranial mass effects, such as nausea, headaches and visual impairment, followed by endocrine disorders, such as amenorrhea, hypogonadism, polyuria and polydipsia (18–20). Furthermore, sexual dysfunction secondary to hypogonadotropic hypogonadism and hyperprolactinemia is the most prevalent endocrine manifestation in adults (17). The hypothalamic damage and mass effects caused by CPs correlate to its anatomical origin and spatial relationship to the hypothalamus (21, 22). CPs that arise in the sellar region are more likely to infiltrate the anterior pituitary tissue, resulting in deficiencies in multiple pituitary hormones (pan-hypopituitarism). In contrast, suprasellar CPs mainly disrupt hypothalamic function, leading to abnormal hypothalamic hormone secretion and pituitary stalk effects, and increased risk of CDI and hyperprolactinemia. Consistent with this, the pre-operative rates of tumor mass effects in the present study were significantly higher in the PCP group, when compared to the ACP group, while suprasellar tumors were more frequent in the PCP group. However, the pre-operative tumor diameter and volume were similar in both groups. Most CP patients present with severe hypothalamic-pituitary dysfunction at diagnosis (23), the extent of which depends on socioeconomic factors, healthcare standards, and disease severity. Nevertheless, gonadal axis dysfunction and hyperprolactinemia are most common, while the adrenal axis is typically the last to be affected. Consistent with this, merely 36.7% of CP patients in the present cohort had intact pre-operative pituitary hormone levels, while 63.3% of the patients presented with at least one neuroendocrine abnormality at diagnosis. Furthermore, hyperprolactinemia was the most prevalent anterior pituitary hormone deficiency, followed by HPG dysfunction, HPT dysfunction, GHD, and HPA dysfunction. However, no significant differences were observed between the ACP and PCP groups.

Almost 54-100% of CP patients develop pituitary hormone deficiencies after surgical intervention (24), with a high prevalence of dysfunctional HPA/HPT/HPG signaling, GH deficiency, and CDI (25). Consistent with this, the deficiencies in ADH, TSH, GH, ACTH, and LH/FSH were exacerbated in both ACP and PCP patients after a median post-operative follow-up of 12 months, when compared to the pre-operative status. In addition, HPA axis impairment, GHD, and pan-hypopituitarism after surgery were more pronounced in the ACP group, when compared to the PCP group. This discrepancy may be attributed to the higher proportion of intrasellar tumors in ACP patients, which increases the risk of surgical damage to the pituitary tissue and function. A retrospective analysis of 500 CP patients who underwent surgery revealed that PCP tumors adhere less to hypothalamic structures, thereby facilitating safer GTR, when compared to ACPs. Karavitaki et al. reported better prognosis of PCPs *vs.* ACPs based on the 10-year endocrine follow-up data of 121 CP patients (26), although other studies have not replicated this finding (27). Recent evidence confirms that unlike pituitary adenomas, the surgical resection of CPs rarely restores pre-existing hormone deficiencies. Furthermore, secondary surgeries and radiotherapy can significantly exacerbate the risk and severity of long-term endocrine hormone deficiencies (28).

Unlike previous studies that have primarily focused on comparing isolated pituitary axis dysfunction in CP patients, the present study quantified the severity of pituitary dysfunction by counting the cumulative number of adenohypophyseal and neurohypophyseal hormone deficiencies. The frequency of post-operative hormone deficiencies was significantly higher in the ACP group, when compared to the PCP group. Furthermore, 4.6% of all CP patients with initially intact hypothalamic-pituitary function developed pan-hypopituitarism after the first surgical intervention. In a study that involved 148 CP patients, pituitary hormone deficiency increased in 63.6% of cases, which is consistent with the present findings. In the same study, transcranial surgery was associated to higher rates of CDI, HPA axis impairment, hypothalamic syndrome, and behavioral abnormalities, when compared to transsphenoidal approaches. However, due to the limited size of the PCP subgroup, the authors did not compare ACP and PCP patients (29). Although surgical resection is presently the preferred treatment for CP, there is ambiguity regarding the appropriate surgical approach, in terms of balancing GTR with the preservation of hypothalamic function, visual outcomes, and QOL (30–33). Elowe-Gruau et al. reported that hypothalamic-sparing surgery is associated with a higher proportion of patients with normal body mass index (BMI) after long-term follow-up, when compared to GTR. However, this did not offer any advantage when tumor recurrence and long-term sequelae associated with adjuvant radiotherapy were considered (34). In another study, CP patients with hypothalamic involvement had reduced 20-year overall survival rates. Notably, the 20-year progression-free survival was independent of the extent of surgical resection, which supports the notion that GTR confers no definitive advantage in preventing CP recurrence (25).

In the present study, multiple intraoperative measures were implemented to preserve pituitary function in CP patients, such as high-definition transnasal endoscopy and 3D endoscopy for enhancing the clarity of the surgical field, intraoperative neurophysiological monitoring and neuronavigation for improving the precision of delineating tumor tissues and its surrounding structures, and endoscopy-assisted compartmentalized resection for preserving the pituitary function in large and highly invasive CPs. Nonetheless, our data indicates that these surgical approaches still exacerbate the degree of pituitary function impairment in CP patients. The surgical approach, GTR rate, tumor recurrence rate, time to recurrence, tumor size at recurrence, and proportion of patients that opted for secondary surgery and radiotherapy upon recurrence were similar in the ACP and PCP groups. A systematic review and meta-analysis conducted by Dandurand et al. revealed no significant differences in tumor recurrence rates among patients who underwent GTR (17%) and subtotal resection followed by radiotherapy (27%) (35). Radiotherapy plays a critical role in the management of CP as an alternative to surgery (36, 37). In the present study, the PCP group had a higher proportion of patients who received radiotherapy, albeit without statistical significance. Recent retrospective studies have indicated that Gamma Knife radiosurgery (GKNS) may be superior to fractionated radiotherapy (FRT) in mitigating endocrine complications (38, 39). Furthermore, Habrand et al. reported cognitive deterioration in 29% of CP patients who underwent FRT, while GKNS was associated with a lower risk (40).

Hoffmann et al. reported that pre-diagnosis disease duration in pediatric CP patients was positively correlated to age and endocrine dysfunction rates, but unrelated to tumor size at diagnosis, extent of resection, hypothalamic involvement, or BMI (41). Furthermore, although some reports suggested higher 5-year overall survival rates for PCP patients, when compared to patients with ACP and mixed histology tumors (27), other reports described an increase in perioperative mortality in patients with adult-onset ACP without confirming the pathological type-specific survival differences (26, 42). We identified tumor histological type, tumor volume, and severity of pre-operative endocrine impairment as risk factors for endocrine outcomes in adult CP patients. Specifically, ACP was linked to a 1.51-fold higher risk of increased post-operative hypothalamic-pituitary impairment, when compared to PCP. Furthermore, patients with normal or mildly impaired pre-operative pituitary function had a higher risk of increased post-operative impairment, thereby confirming the challenges associated with the surgical resection of CPs. Nevertheless, hypothalamic integrity-preserving GTR is the primary goal of present surgical treatment for CPs, and is associated with improved recurrence-free survival rates (43). Long-term QOL and survival in CP patients are closely related to tumor recurrence patterns and therapeutic strategies (44–46). For instance, hypothalamic obesity increases the risk of non-alcoholic steatohepatitis (NASH) by 3- to 9-fold in CP patients (47). Female CP patients face a two-fold higher risk of metabolism-related cardiovascular mortality secondary to hypogonadism, when compared to the general population, which may be attributed to the insufficient attention to GH and gonadal hormone replacement therapy in adult CP patients (14, 48).

Overall, the surgical management of CPs continues to pose challenges in balancing GTR with the preservation of hypothalamic-pituitary function. Further prospective multicenter studies are needed to validate the long-term effects of surgical approaches and resection extent on hypothalamic-pituitary function in CP patients. In addition, standardized assessment of perioperative hypothalamic-pituitary function, long-term post-operative follow-up, and appropriate endocrine hormone replacement therapy would be critical to improving the clinical outcomes of CP patients.

## 5 Conclusion

ACP was the more prevalent histological type of adult-onset CPs, while PCP had a higher propensity for suprasellar growth, leading to greater tumor mass effects and a higher incidence of CDI, when compared to ACP. Surgical intervention significantly increased the spectrum of endocrine hormone deficiencies in both types, and the impact was more pronounced in ACP patients. Furthermore, the ACP histopathology, larger tumor volume, and mild pre-operative endocrine dysfunction were associated with a higher risk of aggravated post-operative hormone deficiencies.

## Data Availability

The original contributions presented in the study are included in the article/supplementary material, further inquiries can be directed to the corresponding author/s.
